# Association between PAPP-A and placental thickness 

**Published:** 2016-06

**Authors:** Elaheh Mesdaghi-nia, Mitra Behrashi, Arezoo Saeidi, Masoomeh Abedzadeh Kalahroodi, Mojtaba Sehat

**Affiliations:** 1 *Kashan University of Medical Sciences, Kashan, Iran.*; 2 *Truma Medical Center, Kashan University of Medical Sciences, Kashan, Iran.*

**Keywords:** *Pregnancy-associated plasma protein-A (PAPP-A)*, *Pregnancy*, *Placenta*

## Abstract

**Background:**

Measuring of maternal serum pregnancy-associated plasma protein-A (PAPP-A) in first trimester can be a way for early detection of adverse prenatal outcome due to faulty placenta.

**Objective:**

The aim was to Determination of association between placental thickness in second trimester with low level of PAPP-A in first trimester.

**Materials and Methods:**

In this cohort study, serum PAPP-A of 187 pregnant women was measured in the first trimester of pregnancy. Patients who had PAPP-A ≤0.8 MOM were in exposed and others who had PAPP-A >0.8 defined as unexposed group. The criteria of placental thickness in ultrasound study was thickness of 4 cm or more than 50% of placental length.

**Results:**

Of 187 patients, 87 patients had PAPP-A >0.8 and 93 patients had PAPP-A ≤0.8. Women with low levels of PAPP-A in the first trimester, had an increased incidence placental thickness of 34.4%, whereas another group had about 15% (p=0.002). Also, PAPP-A levels had acceptable sensitivity and specificity for placental thickness detection (71.1% and 54.8%, respectively.

**Conclusion:**

Our study showed that serum level of PAPP-A generally was low (≤0.8) in women with a thick placenta (>4 cm or >50% of placental length). The first trimester of pregnancy measurement of PAPP-A will be more predictable for healthy placenta.

## Introduction

Pregnancy-associated plasma protein-A (PAPP-A) is synthesized by the placenta and fetus and increases due to pregnancy (1-3). PAPP-A facilitates the actions of the insulin-like growth factor (IGF) family to promote placental growth and function (3-7). Measurement of maternal serum PAPP-A at late in the first trimester of pregnancy in conjunction with maternal age, maternal serum Human Chorionic Gonadotropin (HCG) and fetal nuchal translucency is used to determine the risk of fetal abnormalities such as trisomy 21, with a detection rate of over 90% for a 5% false positive rate (8-11). 

Many studies about chromosomal abnormalities such as trisomy 21, have demonstrated significant correlation between low maternal serum PAPP-A and placental complications in normal pregnancies. Women with PAPP-A below 0.45 MOM (multiples of the median) have a significantly increased risk of intrauterine growth restriction (IUGR), extreme preterm delivery, preeclampsia and stillbirth (8, 12-14). Low PAPP-A levels in the first trimester were associated strongly with a number of adverse pregnancy outcomes (8). Relationship between faulty placenta and adverse prenatal outcome in one hand and observations that suggest measurement of specific circulating trophoblast-derived proteins in the first trimester of pregnancy in other hand may provide a potential screening tool to identify women at increased risk of subsequent adverse pregnancy outcome (12).

Two tests based on ultrasound about placental function (Uterine Artery Doppler (UAD) and measurement of placental size) have been evaluated as screening tests for placental insufficiency in second trimester (16-23 wk) among clinically high-risk women. It was shown that normal placental function profile at 16-23 wk of gestation can reassure women with normal test results by identifying a smaller subset of women who were at reduced risk of prenatal morbidity or death from severe IUGR (15). The prenatal results of abnormal UAD and abnormal placental morphology are similar but performing of UAD require an expert operator and advanced ultrasound device that usually are available in academic centers only (16). 

Considering the limitation of studies on this topic, our study was designed to investigate the possible association between PAPP-A levels in first trimester with placental thickness in pregnant women in order to determine high risk pregnancy based on abnormal placenta. 

## Materials and methods

**Study population**

This cohort study was done to evaluate association between PAPP-A levels in first trimester with placental thickness. Participants were recruited between March to December 2014 from Shahid Beheshti and Shabih Khani hospitals in Kashan, Iran. The study received ethics approval from the ethical committee of Kashan University of Medical Sciences written informed consents were obtained from all participants. All patients who had singleton and low risk pregnancy diagnosed by obstetrician and gynecologist (based on first sonography in pregnancy and first trimester screening test) during this time were included. 

Inclusion criteria were low risk women with less than 14 wk gestational age at first visit and performing first screening test including PAPP-A. Exclusion criteria were presence of fetal anomaly in anatomy scan (done in 18-23 wk), evidence of fetal aneuploidy in first trimester screening test and twin or multiple pregnancy, abortion and not accompaniment of the participants in the study.

**Sample size**

Sample size calculated based on similar study with considering type1 error 0.05 and second type error 20%, P_1_=4.3% and P_2_=15.6% using following formula (16).

$n^{'}={\left[\frac{\left(z_{1-\frac{\alpha }{2}}\sqrt[{2}]{2\bar{p}(1-\bar{p}}\right)+\left(z_{1-\beta }\sqrt[{2}]{p_{1}(1-p_{1}+p_{2}(1-p_{2}}\right)}{p_{1}-p_{2}}\right]}^{2}$

According to the study design and estimate of 20% loss to fallow up, the sample size in each group of samples obtained 90. Based on inclusion criteria and through convenience sampling 187 cases were selected, from them 7 cases were excluded (6 cases due to abortion and one due to twin pregnancy). 

**Measurements**

All subjects that were assessed by first trimester screening test (including PAAP-A with ELISA), followed until 18-23 wk and ultrasound was done by a prenatologist by MEDISON V20 device (11). PAPP-A value ≤0.8 MOM was defined as low level of PAPP-A (8, 12-14). Based on PAPA-A level, patients were divided into two groups: women with PAPA-A ≤0.8 MOM were defined as exposed group and women with PAPA-A >0.8 MOM were defined as unexposed group.

Placental ultrasound assessment was performed between 18-23 wk of gestation, including assessment the placental thickness (15). Ultrasonic thickness of placental was considered when the thickness was 4 cm or more, or was 50% of placental length (16). Demographic characteristics of woman such as age, height and weight to calculate body mass index (BMI), nationality, gestational age, cigarette smoking during pregnancy, history of chronic hypertension and diabetes, parity, history of high risk pregnancy (such as abortion, preeclampsia, placental abruption, preterm labor, intra uterine fetal death (IUFD), IUGR) and method of delivery were recorded in a check list.

**Statistical analysis**

Analysis was done using SPSS Statistics for Windows version 17 (release 17.0.0, Aug 23, 2008) Comparison of outcomes in the two groups was performed using the ^2^ test and placental thickness of the two groups was compared using t-test. Confounding variables were assessed by logistic regression analysis. Specificity and specificity were determined by cut point of 0.8 for PAPP-A value and placental thickness after 8 wk. Statistical significant was considered for p<0.05.

## Results

Mean age of woman was 28.5 (SD:5.7 years and the mean levels of PAPP-A was 0.88±0.51 MOM. Mean age, BMI, history of hypertension, gravidity, parity, live and dead births, abortion and complicated pregnancies were not significantly different between groups (group with PAPP-A ≤0.8 MOM and a group with PAPP-A >0.8) (Table I). Women with PAPP-A ≤0.8 MOM had more placental thickness compared with the other group which was 34.4% in front of 14.9% (p=0.002). The risk of thick placenta in women with low level of PAPP-A was 2.98 fold of the other group (Table II). Moreover the specificity and sensitivity of PAPP-A in placental thickness were obtained 54.8% and 71.1%, respectively, that the percentage of sensitivity was more acceptable (**Table III**). 

The correlation of PAPP-A with the thickness of the placental in pregnant women on the basis of adjusted logistic regression model are presented in table IV. Sensitivity and specificity of PAPP-A at value≤0.8 was 71.1 (55.7-83.6) and 54.8 (46-63.3) respectively. We found Positive predictive value and Negative predictive value of PAPP-A ≤0.8 were 34.4 (24.9-44.9) and 85.1 (75.8-91.8) respectively. Positive Likelihood Ratio with 1.57 was appropriate as a prognostic test.

**Table I:** Characteristics of the study according PAPP-A level

** Groups**		**PAPP-A >0.8 MOM** *	**PAPP-A ≤0.8 MOM**	**p-value**
**Variables**				
Age (years old) **		28.17 (0.65)	28.91 (0.56)	0.385
BMI (kg/cm^2^) **		26.52 (0.38)	27.67 (0.48)	0.06
History of HTN ***		5 (5.7)	8 (9.1)	0.399
Parity **		1.29 (0.54)	1.21 (0.42)	0.455
Abortion ***		15 (68.2)	24 (92.3)	
Gravidity ***		1.87 (0.83)	1.77 (0.81)	0.418
History of complicated pregnancies ***				0.075
	Placental Abruption	1 (4.5)	0	
	Intra Uterine Fetal Death	4 (18.2)	1 (3.8)	
	Intra Uterine Growth Restriction	1 (4.5)	0	
	Preterm labor	1 (4.5)	0	
	Preeclampsia	0	1 (3.8)	

* Multiples of the Median

** Data are presented as Mean (SE)

*** Data are presented as Number (%).

**Table II:** Comparison of placental thickness based on PAPP-A levels

**Placental thickness**	**Yes**		**No**		**RR (95%CI)**	**p-value**
**PAPP-A**	**Number**	**%**	**Number**	**%**		
≤0.8	32	34.4	61	65.6	1.41 (1.03-1.92)	0.04
> 0.8	13	14.9	74	85.1		
Total	45	25	135	75		

RR: Risk Ratio

CI: Confidence interval

**Table IV:** Correlation of PAPP-A with the thickness of the placental in pregnant women on the basis of adjusted logistic regression models

	**Beta**	**SE**	**WALD**	OR***	**Sig.**
PAPP-A>0.8	1.02	0.38	7.1	2.77	0.008
Age	0.04	0.03	1.74	1.05	0.186
BMI	0.049	0.044	1.23	1.05	0.267
Complications of pregnancy	0.091	0.433	0.044	1.1	0.834
HTN	0.477	0.746	0.408	1.61	0.523
constant	-5.36	2.36	5.15	-	0.023

SE: Standard Error

OR: Odds Ratio

## Discussion

This study showed that women with PAPP-A ≤0.8 MoM had more placental thickness compared with other group which was 34.4% and 14.9%, respectively. On the other hand, 85.1% of pregnant woman who had PAPP-A >0.8, did not have placental thickness, which shows having normal level of PAPP-A. Moreover, the specificity and sensitivity of PAPP-A in placental thickness were obtained 54.8% and 71.1%, respectively, that the percentage of sensitivity was more acceptable. 

These findings showed that having normal levels of PAPP-A could be associated with normal pregnancy with low complications. The secretion of PAPP-A in first trimester of pregnancy, is correlated with the placental thickness in second trimester (18-23 wk). PAPP-A is primarily secreted from the placental syncytiotrophoblasts and thought to be involved in normal implantation and placental development (17, 20-22).

In one study, patients were studied four times between 6-13 wk of pregnancy; each time, uterine artery impedance was measured using trans vaginal Color Doppler Ultrasound equipment. They observed that the increasing secretion of PAPP-A negatively correlated with the decreasing uterine artery impedance especially from 10 wk and later, possibly reflecting the rise in blood flow in spiral arteries remodeled by invaded trophoblastic cells. They concluded that failure in trophoblastic function based on low level of PAPP-A can be detected by high flow of UAD (23). 

This finding is similar with our results about correlation of low level of PAPP-A and faulty placenta. In another study, it was shown that the secretion of PAPP-A is closely related to the placenta size in beginning of pregnancy (before 8 wk). The PAPP-A serum concentrations was associated directly with the placental and gestational sac volumes at 5-8 wk of pregnancy. The change in placental volume between 5-8 wk was correlated with the change in the PAPP-A serum concentrations at the corresponding time (24). 

However, their study have two differences with our study: 1) measurement of placental and gestational sac volumes instead of placental thickness. 2) evaluating at 5-8 wk of pregnancy instead of 18-23 wk gestation. But they proved our results while the mentioned study showed this correlation (between PAPP-A and placental size) earlier than our study. Another study on pregnant women in first trimester, showed that low level of PAPP-A in this time is a predictive factor of adverse pregnancy outcome (as fetal grows restriction (FGR), pregnancy associated hypertension and spontaneous abortion) (25). Ranta *et al* showed that the low level of PAPP-A in first trimester is related with preeclampsia, preterm labor and small for gestational age (SGA) (26). 

Other studies have shown that abnormal levels of PAPP-A (low level) during first trimester of pregnancy seems to be capable of predicting adverse outcomes such as IUGR, preeclampsia or preterm delivery taking place later in the pregnancy (27-29). This explains relationship between abnormal placental morphology and adverse prenatal outcomes that’s similar to our study.

According to high rate of negative predictive value of PAPP-A, it can be concluded that normal PAPP-A (>0.8) is a good predictor of health status of placenta in later months. In summary, since circulating concentrations of PAPP-A had inverse correlation with the thickness of the placenta and its sensitivity was high, it remains to be shown that adverse pregnancy outcomes can be predicted by measuring placental thickness which had inverse relationship with PAPP-A during the first trimester of pregnancy.

This is a unique study that is trying to get placental insufficiency based on placental thickness in second trimester with measuring of PAPP-A in many time earlier in first trimester. This study is the first study to evaluate the relationship between the placental thickness and the secretion of PAPP-A. 

**Limitation**

Limitations of this study is lack of evaluation of adverse pregnancy outcomes in samples at the same time. But it is followed a way to inform the physician earlier in order to alarm about a high risk pregnancy that may be caused by a thick or insufficient placenta.

## Conclusion

Result of this study showed mean thicknesses of placenta in women with low level of PAPP-A (≤0.8) is high (>4 cm or >50% of placental length). our data also showd that the sensitivity of PAPP-A in placental thickness was obtained 71.1% that more acceptable against specificity 54.8%. In other words, normal PAPP-A (>0.8) in first trimester showed that there was normal placental thickness in 85.1% of cases while low level of PAPP-A only in 34.4% showed thick placenta. PAPP-A level in first trimester have more applications in predicting normal placenta, whereas low PAPP-A does not haven great value in predicting thick placenta. 
